# Network Pharmacology Combined with Bioinformatics to Investigate the Mechanisms and Molecular Targets of *Astragalus Radix-Panax notoginseng* Herb Pair on Treating Diabetic Nephropathy

**DOI:** 10.1155/2021/9980981

**Published:** 2021-07-24

**Authors:** Jie Zhao, Chao Mo, Wei Shi, LiFeng Meng, Jun Ai

**Affiliations:** ^1^Graduate School, Hunan University of Chinese Medicine, Changsha, Hunan 410208, China; ^2^Department of Nephrology, The First Affiliated Hospital of Guangxi University of Chinese Medicine, Nanning, Guangxi 530023, China; ^3^Traditional Chinese and Western Medicine Laboratory for Controlling Organ Fibrosis, Southwest Medical University, Luzhou, Sichuan 646000, China; ^4^Basic Medicine School, Guangxi University of Chinese Medicine, Nanning, Guangxi 530200, China

## Abstract

**Background:**

*Astragalus Radix* (AR)-*Panax notoginseng* (PN), a classical herb pair, has shown significant effects in treating diabetic nephropathy (DN). However, the intrinsic mechanism of *AR-PN* treating DN is still unclear. This study aims to illustrate the mechanism and molecular targets of *AR*-*PN* treating DN based on network pharmacology combined with bioinformatics.

**Materials and Methods:**

The Traditional Chinese Medicine Systems Pharmacology database was used to screen bioactive ingredients of *AR*-*PN*. Subsequently, putative targets of bioactive ingredients were predicted utilizing the DrugBank database and converted into genes on UniProtKB database. DN-related targets were retrieved via analyzing published microarray data (GSE30528) from the Gene Expression Omnibus database. Protein-protein interaction networks of *AR*-*PN* putative targets and DN-related targets were established to identify candidate targets using Cytoscape 3.8.0. GO and KEGG enrichment analyses of candidate targets were reflected using a plugin ClueGO of Cytoscape. Molecular docking was performed using AutoDock Vina software, and the results were visualized by Pymol software. The diagnostic capacity of hub genes was verified by receiver operating characteristic (ROC) curves.

**Results:**

Twenty-two bioactive ingredients and 189 putative targets of *AR-PN* were obtained. Eight hundred and fifty differently expressed genes related to DN were screened. The PPI network showed that 115 candidate targets of *AR-PN* against DN were identified. GO and KEGG analyses revealed that candidate targets of *AR-PN* against DN were mainly involved in the apoptosis, oxidative stress, cell cycle, and inflammation response, regulating the PI3K-Akt signaling pathway, cell cycle, and MAPK signaling pathway. Moreover, MAPK1, AKT1, GSK3B, CDKN1A, TP53, RELA, MYC, GRB2, JUN, and EGFR were considered as the core potential therapeutic targets. Molecular docking demonstrated that these core targets had a great binding affinity with quercetin, kaempferol, isorhamnetin, and formononetin components. ROC curve analysis showed that AKT1, TP53, RELA, JUN, CDKN1A, and EGFR are effective in discriminating DN from controls.

**Conclusions:**

*AR-PN* against DN may exert its renoprotective effects via various bioactive chemicals and the related pharmacological pathways, involving multiple molecular targets, which may be a promising herb pair treating DN. Nevertheless, these results should be further validated by experimental evidence.

## 1. Introduction

Diabetes mellitus (DM) is a common metabolic disorder characterized by chronic hyperglycemia due to defective insulin secretion and/or utilization, which has been considered an enormous global health concern [1]. According to the International Diabetes Federation data, 352 million people between the ages of 20 and 65 years are suffering from DM worldwide, which will occur in up to 486 million by 2045 [2]. Diabetic nephropathy (DN), a major microvascular complication of DM, is a leading cause of end-stage renal disease [3]. Research studies indicate that 20%–40% of patients suffering from DM develop DN and patients with DN have a higher risk of death with 3 to 12 times than those with DM alone [4, 5]. Advances have been made in the treatment of DN in recent years with the introduction of lifestyle changes, including exercise and diet, and therapies such as angiotensin-converting enzyme inhibitors, angiotensin receptor blockers, sodium-glucose cotransporter 2 inhibitors, and glucagon-like peptide-1 receptor [3, 6]. Unfortunately, these drugs were reported to have adverse effects, such as diabetic ketoacidosis, gastrointestinal, and hepatotoxicity [7, 8], and there are still no pharmacotherapies available to cure or reverse disease progression. Highlighting the requirement for novel therapeutic with better efficacy and safe treating DN is still necessary.

Chinese herbal medicine with various bioactive chemicals and the related pharmacological pathways, involving multiple cellular and molecular targets, and fewer side effects has been frequently and extensively used for thousands of years in China [9], which may be to provide beneficial and effective therapy for DN. According to the theory of traditional Chinese medicine (TCM), the basic pathology of DN is qi deficiency and blood stasis [10]. Hence, tonifying qi and activating blood circulation are the basic therapeutic principles for DN. *Astragalus Radix* (*AR*, Huang qi in Chinese) was initially recorded in *Shennong Bencao Jing*, which has been extensively known to exert the tonifying Qi effect. *Panax notoginseng* (*PN*, San qi in Chinese) was found in *Bencao Gangmu* with multiple beneficial effects, especially activating blood circulation and decreasing swelling to relieving pain. The herb pair is considered the most basic form of TCM therapy containing two special Chinese herbal medicines, which plays a vital role in connecting mutual enhancement, assistance, and restraint [11]. *AR*-*PN*, a classic highly valued and widely used herb pair, is well known to possess functions with tonifying qi and activating blood circulation. Modern pharmacological research shows that *AR* treatment ameliorated the severity of DN by inhibiting inflammation-related gene IL-1*β* and IL-18 expression and fibrosis indexes in DN rats [12]. Likewise, it has been confirmed that *AR* possesses antioxidative stress effects [13]. Previous research demonstrated that *PN* can significantly reduce albuminuria, proteinuria, and serum creatinine and improve the metabolism of serum lipids in patients with DN [14]. In addition, both in vivo and in vitro research studies confirmed that the combination of *AR* and *PN* could strengthen the renoprotection effects by inhibiting inflammation, alleviating oxidative stress, and upregulating autophagy [15]. Taken together, the *AR-PN* herb pair playing a renoprotection role in DN is mainly aimed at anti-inflammation and antioxidative stress. However, the underlying pharmacological mechanism of the *AR-PN* herb pair in the treatment of DN remains to be illustrated. A novel approach to clarify the pharmacological mechanisms of the efficacy of the *AR-PN* herb pair on DN is necessary.

Network pharmacology was first proposed by Hopkins in 2007, which provides a convenient and systematic method to reveal the complex mechanisms of drug treating disease through identifying core targets based on the theory of systems biology and network pharmacology [16]. Moreover, network pharmacology is also contributed to integrate and extract possible signaling pathways that drugs prevent and treat diseases to improve the therapeutic effect of drugs, and the pattern is “drug-target-disease.” It has been applied to explore the mechanisms of TCM treating some diseases, including diabetes mellitus [17] and ischemic stroke [18], showing that applying network pharmacology to elucidate the mechanisms of bioactive components and the potential of TCM is an emerging trend. Bioinformatics is the application of tools of computation and analysis to the capture and interpretation of biological data, which is an interdisciplinary field of computer science, mathematics, physics, and biology [19]. Currently, bioinformatics has been increasingly recommended in the study of biological fields, particularly in genomics and proteomics. miRNAs involve the fine-tuning of mRNA abundance via binding to the 3′ untranslated region of a target mRNA and results in translational repression. Increasing evidence suggests that the downregulation or upregulation of miRNAs is closely associated with podocyte injury, inflammation, accumulation of the extracellular matrix, fibrosis, and so on in DN, concerning their potential as biomarkers and miRNA modulation as a therapeutic option for DN [20, 21]. Besides, some microarray data analysis studies related to DN have been performed and numerous DEGs have been identified, which may be potential biomarkers and target candidates in DN [22]. Except for DN, bioinformatics strategies are also involved in identifying the core genes of other diseases, such as diabesity [23], familial hypercholesterolemia [24], lung squamous cell carcinoma [25], and prostate cancer [26], indicating that bioinformatics plays vital roles in predicting potential biomarkers and providing therapeutic options. Collectively, network pharmacology integrated with bioinformatics is a novel strategy to study ingredient identification, target prediction, and therapeutic options.

**Figure 1:**
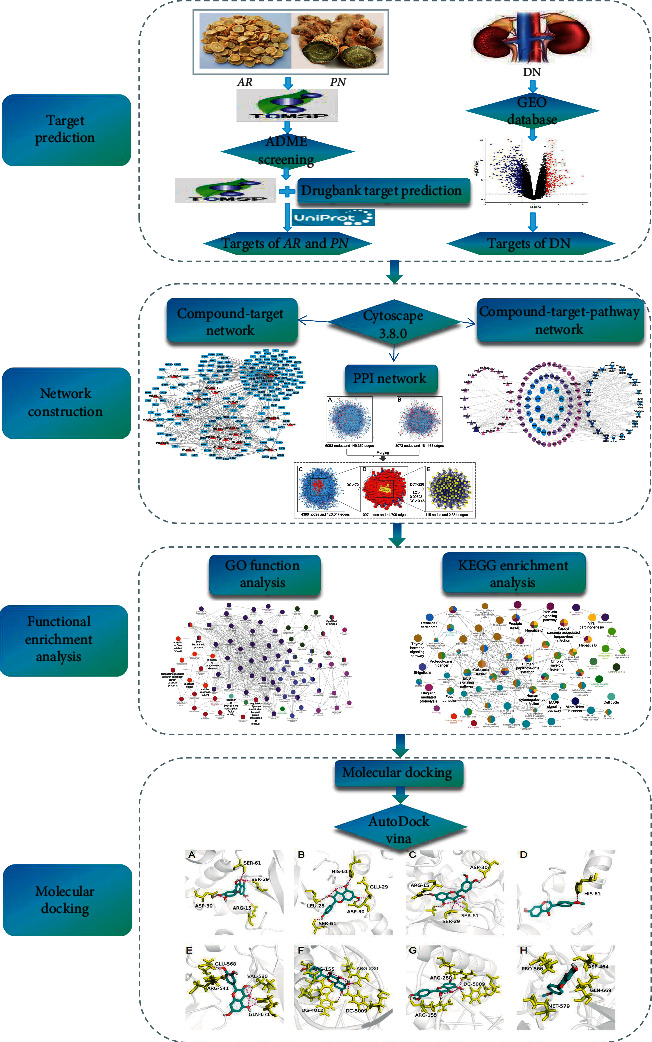
The flowchart of network pharmacology study. *AR*: *Astragalus Radix*; *PN*: *Panax notoginseng*; DN: diabetic nephropathy.

Consequently, we integrated network pharmacology with Gene Expression Omnibus (GEO) database to further identify the comprehensive pharmacological mechanisms, screen for potential molecular targets, and explore underlying pathways of *AR-PN* herb pair on treating DN, which may provide a guideline in the further research and development. The flowchart of this study is shown in Figure 1.

## 2. Material and Methods

### 2.1. Screening of Active Ingredients of *AR*-*PN* Herb Pair

All active ingredients of the *AR-PN* herb pair were collected from the Traditional Chinese Medicine Systems Pharmacology (TCMSP) database (http://tcmspw.com/tcmsp.php), which is a unique systematic pharmacology platform to display the relationships between components, targets, and diseases [27]. The absorption, distribution, metabolism, and excretion (ADME) model [28], which was an important contributor to predict the pharmacokinetic properties of chemical ingredients, was used to screen the compounds of the *AR-PN* herb pair. Two vital parameters, i.e., oral bioavailability (OB) and druglikeness (DL), among all ADME-related properties were used to identify chemical ingredients of the *AR-PN* herb pair. The OB refers to the relative amount of a drug absorbed into the systemic circulation after extravascular administration [29]. DL is used to estimate the similitude physiochemical properties or/and structural features between an ingredient and certified drug candidates [30], which is calculated by the following formula:

(1) $$\begin{array}{c} \text{DL }\left(X,\;Y\right)=\frac{X\times Y}{\left|X^{2}\right|+\left|Y^{2}\right|-X\times Y}, \end{array}$$

where “*X*” represents the active ingredients of the *AR-PN* herb pair and “*Y*” stands for the average DL index of all molecules that are collected from the DrugBank database (https://www.drugbank.ca) [31]. In our present study, bioactive compounds were collected according to the threshold values of OB ≥ 30% and DL ≥ 0.18.

### 2.2. Prediction of the *AR-PN* Herb Pair-Related Targets

Identification of putative targets of the *AR-PN* herb pair bioactive ingredients was performed with DrugBank. Subsequently, all proteins of each bioactive ingredient of the *AR-PN* herb pair were converted into genes in the UniProt database (http://www.uniprot.org/) [32] with species as “*Homo sapiens*” limited. Ultimately, all putative targets of the *AR-PN* herb pair were obtained after removing duplicated targets. Moreover, we used Cytoscape 3.8.0 software (http://www.cytoscape.org/) to establish and visualize the component-target network of the *AR-PN* herb pair.

### 2.3. Differentially Expressed Gene Search, Identification, and Analysis of DN

Expression profiling data and Raw CEL data from the GSE30528 dataset [33] were acquired from the GEO database according to Affymetrix GPL570 platform and Affymetrix Human Genome U133 Plus 2.0 Array, which included nine samples from DN glomeruli tissues and 13 samples from controlled normal glomeruli tissues. Then, probe IDs were applied to identify the corresponding genes. Subsequently, differentially expressed genes (DEGs) between DN glomeruli tissues and controlled normal glomeruli tissues were screened by the limma package of R software. DEGs with *P* < 0.05 and |log2 fold change (FC)| > 1 were selected as genes of DN and were visualized using a volcano plot.

### 2.4. Construction of Protein-Protein Interaction Network

Protein-protein interaction (PPI) is the core of almost all biological processes, which present the interaction of proteins and may provide potential targets for therapeutic intervention [34]. The PPI networks of the *AR-PN* herb pair putative targets as well as DN-related DEGs were constructed and visualized using the BisoGenet [35], a plugin of Cytoscape 3.8.0, which were established based on the currently available PPI databases, including Biological General Repository for Interaction Datasets (BioGRID), Database of Interacting Proteins (DIP), Biomolecular Interaction Network Database (BIND), IntAct Molecular Interaction Database (IntAct), Human Protein Reference Database (HPRD), and Molecular INTeraction Database (MINT). Subsequently, a merged network was performed according to the intersection data of the two networks.

### 2.5. Network Topological Characteristics Analysis

The three crucial topological properties, including betweenness centrality (BC), closeness centrality (CC), and degree centrality (DC), were used to calculate and analyze topological properties of every node in the merged interaction network using the CytoNCA [36], a plugin of Cytoscape 3.8.0. The node is more important, and the received quantitative value is higher. First, the degree of centrality was calculated and the network was extracted according to more than twice the median degree of DC. Then, more than the median values of DC, BC, and CC as the threshold values were used to further extract hub nodes in the network. Ultimately, a core subnetwork was obtained for the ensuing analysis.

### 2.6. GO and KEGG Pathway Enrichment Analysis

GO, a freely available public resource, provides a standardized language to systematically describe the functional properties of gene products from all species, which consists of three ontologies, namely, biological process (BP), cellular component (CC), and molecular functional (MF) [37]. BP describes the functioning of biological objectives to which they contribute, CC describes the place of a gene product within the cell and their extracellular environments, and MF represents the biochemical activities, such as binding and catalysis, of gene products [38]. KEGG is a highly complex network structure, which has been an exceptionally comprehensive site for describing metabolic pathways, gene signaling networks, and practical application of genomic information [39]. The biological interpretations of the hub genes in the core network were reflected by GO and KEGG pathway enrichment analysis using ClueGO, a plugin of Cytoscape 3.8.0, which is utilized to visualize nonrepetitive biological terms as functionally grouped networks. It is worth noting that the *P* value less than or equal to 0.01 was set as the threshold in both the GO or KEGG functional categories. Furthermore, the component-target-pathway network was established and visualized using Cytoscape 3.8.0 software.

### 2.7. Molecular Docking

Molecular docking, a method of analyzing the binding site and affinity between the conformation of molecules and macromolecular targets, has been considered a crucial technique for structure-based drug discovery [40]. The core DN-related genes targeted by the *AR-PN* herb pair bioactive components was identified according to the component-target-pathway network, which were further validated by molecular docking with the experimentally verified *AR-PN* herb pair bioactive components possessing renoprotective effects on DN, including quercetin [41], kaempferol [42], formononetin [43], and isorhamnetin [44]. First, the mol2 formats of these bioactive components were obtained from the TCMSP database and PubChem database, which were converted to the format of pdb and pdbqt via Pymol software and AutoDock Tools 1.5.6, respectively. Consequently, the 3-dimensional structures of potential receptors saved in pdb format were downloaded from the PDB database (https://www.wwpdb.org/). The removal of the ligand and water in the protein was performed via AutoDock Tools 1.5.6., which was saved in pdbqt format. Subsequently, molecular docking was performed to evaluate the combination mode and affinity between the ligand-receptor and molecules by AutoDock Vina, which was run with default settings. The smaller the lowest energy value is, the more stable and the stronger affinity between ligand and receptor binding. Ultimately, we selected the top two receptor proteins with the lowest energy value and the ligand for visualization and construction by Pymol software.

### 2.8. Receiver Operating Characteristic (ROC) Curves for Hub Genes

To evaluate the diagnostic accuracy of each hub gene for DN, ROC curves were plotted and area under the curve (AUC) values were calculated, which were performed with “pROC” package of R software 4.0.3. *P* < 0.05 was considered to be significant.

## 3. Results

### 3.1. Screening of Bioactive Components and Putative Targets of the *AR-PN* Herb Pair

**Figure 2:**
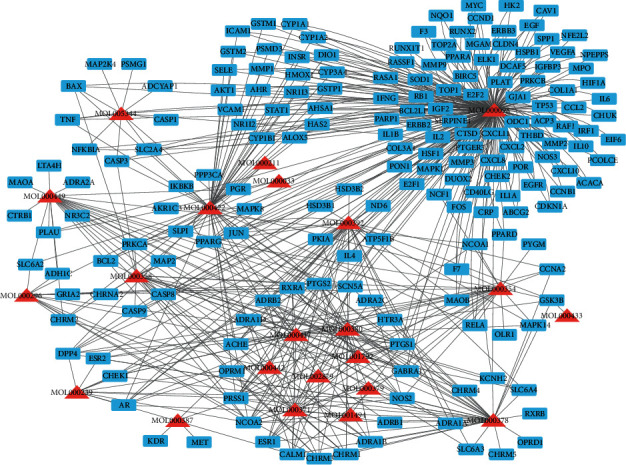
Compound-target network of *AR-PN* herb pair. Blue squares represent targets, and red triangles represent ingredients of *AR*-*PN* herb pair.

A total of 27 bioactive components, including 19 components in *AR*, seven components in *PN*, and one component in both *AR* and *PN*, were identified which met the criterion of OB ≥ 30% and DL ≥ 0.18 as the threshold from the TCMSP database. The information of bioactive components of *AR*-*PN* herb pair is shown in Table 1. Five bioactive ingredients without any corresponding target, whose mol IDs were MOL000374, MOL000398, MOL000438, MOL000439, and MOL007475, were excluded. A total of 461 target proteins and 252 target proteins of *AR* and *PN* active components were collected, respectively. After deleting duplicate targets, 189 putative targets of *AR-PN* herb pair were identified. The compound-target network of *AR-PN* herb pair consisted of 211 nodes and 583 edges, which is shown in Figure 2.

### 3.2. Identification of DN-Related DEGs

**Figure 3:**
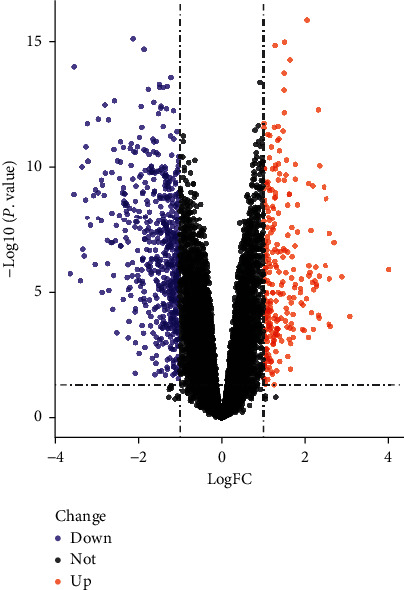
A volcano plot of differentially expressed genes of DN. The red dots represent significantly upregulated genes, and the blue dots represent significantly downregulated genes.

Ultimately, 850 DN-related DEGs were identified between DN glomeruli tissues and controlled normal glomeruli tissues with |log2 FC| > 1 and *P* < 0.05 by analyzing the sequencing data downloaded from the GEO database. A volcano plot of the distribution of DEGs is exhibited in Figure 3, which includes 253 upregulated genes dotted in red, 597 downregulated genes dotted in blue, and other genes without significant differences dotted in black.

### 3.3. PPI Network Construction, Merging, and Topology Analysis

**Figure 4:**
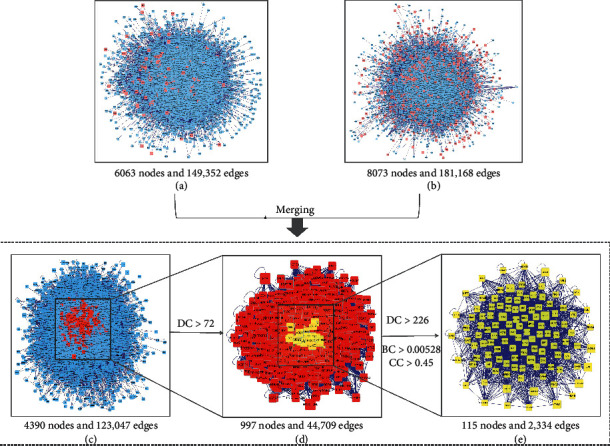
Protein-protein interaction (PPI) network construction and topology analysis. (a) The PPI network of *AR-PN* herb pair putative targets. (b) The PPI network of DN-related DEGs. (c) The interactive PPI network of *AR-PN* herb pair putative targets and DN-related DEGs. (d) The PPI network of significant proteins screened from (c). (e) The PPI network of candidate targets of *AR-PN* herb pair against DN screened from (d). BC: betweenness centrality; CC: closeness centrality; DC: degree centrality; DEGs: differentially expressed genes.

To illustrate the interactions and underlying mechanisms between *AR-PN* herb pair putative targets and DN-related DEGs, we visually constructed PPI networks of *AR-PN* herb pair putative targets and DN-related DEGs, respectively. The PPI network of *AR-PN* herb pair putative targets contained 6063 nodes and 149,352 edges (Figure 4(a)), while the PPI network of DN-related DEGs contained 8073 nodes and 181,168 edges (Figure 4(b)). In the PPI network graph, nodes represent interacting proteins, and edges represent interactions. A new PPI network was generated after merging these two PPI networks to identify the candidate targets for the *AR-PN* herb pair in the treatment of DN, which comprised 4390 nodes and 123,047 edges (Figure 4(c)). Subsequently, we analyzed the topological properties of the merged PPI network based on BC, CC, and DC key parameters and extracted targets above twofold median values of DC as well as more than the median values of BC and CC. Therefore, nodes with DC that were above twofold median values (DC > 72) of all nodes were the first extracted, which included 997 nodes and 44,709 edges (Figure 4(d)). Whereafter, nodes with DC > 226, BC > 0.000528, and CC > 0.45 of these 997 nodes were the second identified core targets containing 115 nodes and 2,334 edges (Figure 4(e)). The information of 115 candidate targets sorted in descending order based on the value of the degree is presented in Table 2.

### 3.4. GO and KEGG Pathway Enrichment Analysis

**Figure 5:**
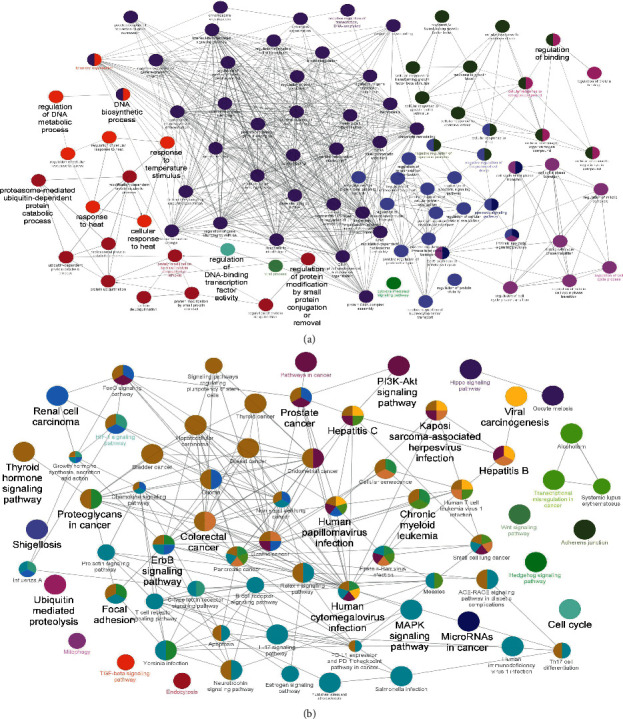
Enrichment analysis of candidate targets based on ClueGO. (a) Possible enriched targets of *AR-PN* herb pair in typical biological processes. (b) Possible enriched targets of *AR-PN* herb pair in signaling pathways.

To further evaluate better the molecular mechanism of the *AR-PN* herb pair on DN, GO and KEGG pathway enrichment analyses associated with 115 candidate targets were performed using the Cytoscape plugin ClueGO, which included GO biological process and the signaling pathway. The GO biological processes were mainly involved in regulating DNA-binding transcription factor activity, regulating protein modification by small protein conjugation or removal, rhythmic process, proteasome-mediated ubiquitin-dependent protein catabolic process, and cellular response to heat, and so on (Figure 5(a)). According to the KEGG pathway analysis, the signaling pathways were mainly focused on the PI3K-Akt signaling pathway, viral carcinogenesis, cell cycle, microRNAs in cancer, and hepatitis B, and so on (Figure 5(b)).

### 3.5. Construction of the Component-Target-Pathway Network

**Figure 6:**
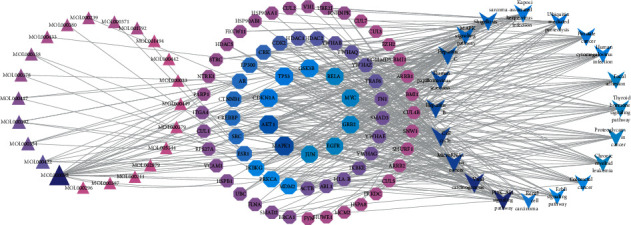
Component-target-pathway network of *AR-PN* herb pair against DN. The larger size and darker the color of nodes were shown, the higher the degree value was represented. Rhombus represents targets, triangles represent ingredients of *AR-PN* herb pair, and arrowhead represents signal pathways.

To further explore the interaction between components, candidate targets, and the top 20 signaling pathways, the network map of component-target-pathway was constructed by Cytoscape 3.8.0, which included 113 nodes and 395 edges (Figure 6). The larger and darker the color of nodes was shown, the higher the degree value was represented, the thicker the edges were displayed, and the greater closeness and betweenness were found. Hence, we speculated that quercetin (MOL000098), kaempferol (MOL000422), isorhamnetin (MOL000354), and formononetin (MOL000392) regulate the PI3K-Akt signaling pathway, viral carcinogenesis, microRNAs in cancer, cell cycle, and MAPK signaling pathway, and so on, via core target genes MAPK1 (PDB ID: 4iz5), AKT1 (PDB ID: 1unq), GSK3B (PDB ID: 4afj), CDKN1A (PDB ID: 2zvw), TP53 (PDB ID: 2k8f), RELA (PDB ID: 1nfi), MYC (PDB ID: 5g1x), GRB2 (PDB ID: 1gri), JUN (PDB ID: 1s9k), and EGFR (PDB ID: 5wb7).

### 3.6. Molecular Docking Analysis

**Figure 7:**
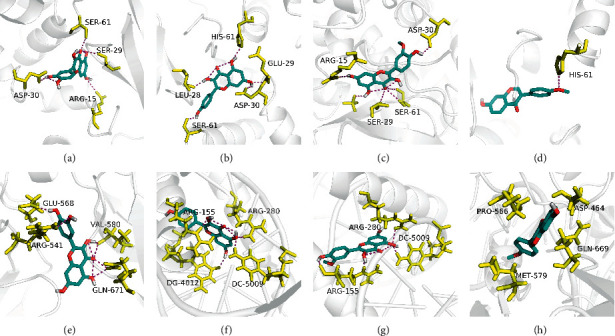
Molecular models of the binding of quercetin, kaempferol, isorhamnetin, and formononetin with MAPK1 (a–d) and JUN (e–h), respectively.

According to the network of component-target-pathway, the core targets (MAPK1, AKT1, GSK3B, CDKN1A, TP53, RELA, MYC, GRB2, JUN, and EGFR) were selected for molecular docking with quercetin, kaempferol, isorhamnetin, and formononetin bioactive components verified experimentally via AutoDock Vina 1.1.2 software. As shown in Table 3, all the core targets mentioned above had a great binding affinity with quercetin, kaempferol, isorhamnetin, and formononetin. Furthermore, the MAPK1 and JUN target proteins were molecularly docked with quercetin, kaempferol, isorhamnetin, and formononetin active components with the lowest energy value that was less than –8.0 kcal·mol^−1^. Therefore, we selected the MAPK1 and JUN receptor proteins with the lowest energy value and the ligands for visualization and construction by Pymol software (Figure 7).

### 3.7. Verification of Hub Genes by ROC Curve Analysis

**Figure 8:**
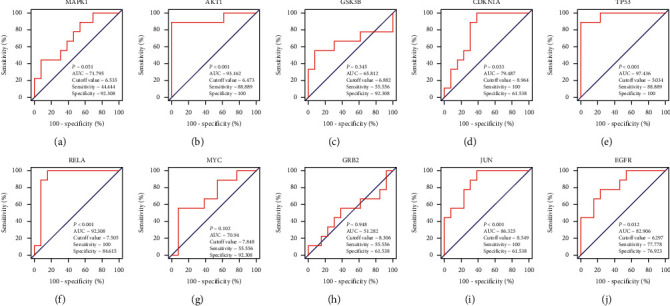
Receiver operating characteristic curves of hub genes, including MAPK1, AKT1, GSK3B, CDKN1A, TP53, RELA, MYC, GRB2, JUN, and EGFR in the GSE30528 dataset.

ROC curves were applied to evaluate the diagnostic value of the hub genes for DN. This analysis showed that AKT1 (AUC = 0.93162; *P* < 0.001), TP53 (AUC = 0.97436; *P* < 0.001), RELA (AUC = 0.92308; *P* < 0.001), JUN (AUC = 0.86325; *P* < 0.001), CDKN1A (AUC = 0.79487; *P* = 0.033), and EGFR (AUC = 0.82906; *P* = 0.012) are effective in discriminating between DN patients and controls in the GSE30528 dataset. However, MAPK1 (AUC = 0.71795; *P* = 0.051), GSK3B (AUC = 0.65812; *P* = 0.345), MYC (AUC = 0.70940; *P* = 0.102), and GRB2 (AUC = 0.51282; *P* = 0.948) were proved to be no diagnostic capability for DN (Figure 8).

## 4. Discussion

Microarrays are a powerful and effective high-throughput technology and provide information on gene profiling data to researchers to explore biomarker, molecular pathways, target selectivity, and development of prognostic, and so on involved in complex disorders [45]. They have been widely applied to investigate the key molecule biomarkers and pathways of various diseases, including ovarian cancer [46], multiple myeloma [47], systemic lupus erythematosus (SLE) [48], and idiopathic pulmonary fibrosis [49]. DN is the most common microvascular complication of DM, which has been considered the major cause of ESRD worldwide [3]. Nevertheless, effective treatments remain scarce. Previous studies have been demonstrated that *AR* and *PN* play a critical role in treating DN [12–14, 50], suggesting *AR-PN* may be a potential herb pair for the treatment of DN. For all we know, this is the first study to perform a comprehensive analysis of network pharmacology combined with gene expression profiling to further explore the underlying pharmacological mechanisms and therapeutic targets of the *AR-PN* herb pair on DN. Our findings demonstrated that a total of 22 bioactive compounds and 115 core targets for the *AR-PN* herb pair in the treatment of DN were identified. According to the KEGG pathway enrichment, the *AR-PN* herb pair played a therapeutic role in DN through regulating the PI3K-Akt signaling pathway, cell cycle, and MAPK signaling pathway, and so on. Furthermore, MAPK1, AKT1, GSK3B, CDKN1A, TP53, RELA, MYC, GRB2, JUN, and EGFR were regarded as core targets of the *AR-PN* herb pair against DN according to the component-target-pathway network. What is more, molecular docking analysis validated that these core target genes showed strong binding interactions and affinity with quercetin, kaempferol, isorhamnetin, and formononetin bioactive compounds of the *AR-PN* herb pair.

As shown in the component-target network (Figure 2), 22 compounds in the *AR-PN* herb pair with possible efficacy against DN were identified, many of which shared targets and had a synergistic effect, indicating that the *AR-PN* herb pair exerts effects in various bioactive components and multiple cellulars and molecular targets way. Quercetin, a natural flavonoid, is well known to possess multiple pharmacological functions, including but not limited to antioxidant, anti-inflammatory, anticancer, antidiabetic, neuroprotective, and antiobesity [51, 52]. The use of quercetin contributes to a meaningful improvement in disease symptoms, renal hypertrophy index, renal histopathology, oxidative stress, and blood glucose in a rat model of DN [41]. Furthermore, quercetin treatment is beneficial to antioxidant capacity and vasoprotective effects against STZ-induced ER stress of diabetic rats via reducing the lipid peroxidation and increasing the expression of vascular endothelial growth factor (VEGF) and its receptor, VEGFR2, and nitric oxide (NO) [53]. Accumulating evidence also demonstrated that the nephroprotective effects of quercetin are related to inhibiting renal tubular epithelial-mesenchymal transition and renal fibrosis and reducing the production of cytokines in DN, including IL-6, TNF-*α*, and IL-1*β* [54–56]. What is more, quercetin inhibited glomerular mesangial cell proliferation and epithelial-mesenchymal transition in DN via reactivation of the Hippo pathway and inhibition of the TGF-*β*/PI3K/Akt pathway, respectively [57, 58]. Kaempferol, a natural polyflavonol, also exhibits nephroprotective effects in addition to its antioxidant and anti-inflammatory properties [59]. Studies demonstrated that kaempferol plays vital nephroprotective roles associating with inhibiting oxidative stress, proinflammatory cytokines (TNF-*α* and IL-1*β*), and fibrosis in DN via inhibiting hyperglycemia-induced activation RhoA/Rho-kinase [42]. Moreover, kaempferol exerts antioxidant potential in DN rats by the upregulation of the Nrf-2/HO-1 axis [60]. Formononetin possesses multiple biological activities, such as antioxidant, anti-inflammatory, and regulating immune responses [61, 62]. Formononetin improved oxidative stress and prevented the progression of renal fibrosis in DN by activating the Nrf2/ARE signaling pathway [43]. Experimental studies have shown that isorhamnetin has renoprotective effects on DN through improving fasting blood glucose, increasing autophagosomes in renal tissues, and suppressing miRNA regulation of autophagy genes [44]. Besides, isorhamnetin inhibits oxidative stress, inflammation, and apoptosis in STZ-induced diabetic rats [63]. Considering this, quercetin, kaempferol, formononetin, and isorhamnetin are the potential components of the *AR-PN* herb pair treating DN, associated with their various bioactive actions, such as antioxidant, anti-inflammation, and antiapoptosis.

Subsequently, 115 nodes of the *AR-PN* herb pair acting on DN were identified based on the merger PPI network, which were considered as core targets that the *AR-PN* herb pair plays therapeutic roles in DN. To further illustrate the mechanism of *AR-PN* herb pair on DN, we conducted GO and KEGG pathway enrichment analyses of 115 core targets. As shown in Figure 5(a), the BP terms of *AR-PN* herb pair on DN were mainly related to DNA synthesis, transcription, protein catabolism, cell cycle, apoptosis, oxidative stress, and inflammation regulation. Interestingly, apoptosis, oxidative stress, and inflammation are deemed as the important pathogenesis of DN [3]. KEGG enrichment analysis showed that the *AR-PN* herb pair may alleviate the development of DN through the action of core targets in multiple signaling pathways (Figure 5(b)), including PI3K-Akt signaling pathway, cell cycle, and MAPK signaling pathway. Among them, the enriched targets of the PI3K-Akt signaling pathway are markedly higher than others. Phosphoinositide-3-kinase (PI3K) can promote the phosphorylation of protein kinase B (PKB, Akt), which involves diversified cell activities, e.g., cell proliferation, protein synthesis, glycolysis, autophagy, and apoptosis [64]. Previous studies demonstrated that restoring podocyte autophagy, attenuating podocyte apoptosis, and inhibiting MC proliferation of DN were closely associated with inhibiting the PI3K/AKT signaling pathway [65–67]. Furthermore, notoginsenoside R1, which is extracted from *PN*, increased the phosphorylation of both PI3K and Akt and ameliorated podocyte injury in DN rats by activating the PI3K/Akt signaling pathway [68]. Cell cycle refers to order progression from one cell cycle phase to the next, involving a family of cyclins and their associated kinases, and mediates cell proliferation, which will be ceased if its checkpoint secures, such as Rad3-related protein kinase and checkpoint kinase 1, causing severe problems [69]. Moreover, the cell cycle is regulated by several cellular pathways, e.g., PI3K/Akt signaling pathway that is famous for regulating cell proliferation [64]. MAPK pathway acts on inhibiting cell apoptosis and promoting proliferation and anti-inflammatory effects in DN rats, which is associated with MAPK1 [70]. Viral carcinogenesis accelerates DNA damage and virus integration, which are closely related to the progression of human cancers [71], as well as microRNAs in cancer [72], suggesting *AR-PN* herb pair also exerts anticancer effects. Thus, our identified core targets are highly related to the signaling pathways associated with cell proliferation, apoptosis, and cell cycle.

Furthermore, the network of component-target-pathway showed that MAPK1, AKT1, GSK3B, CDKN1A, TP53, RELA, MYC, GRB2, JUN, and EGFR were core targets in the network due to possessing the higher degree (Figure 6), suggesting that these core targets were identified as core genes that were strongly associated with the pathogenesis of *AR-PN* herb against DN. MAPK1, also known as an extracellular signal-regulated protein kinase, is a member of the MAPKs family that is contributed to enhancing cellular proliferation and participating in cell metabolism, proliferation, apoptosis, differentiation, and survival in physiological and pathological processes [73]. It has been demonstrated that downregulated expression of MAPK1 plays a protective role in lipopolysaccharide-induced podocyte damage in DN [74]. Many studies have observed that the expression of MAPK1 was increased in various cancers, such as breast cancer [75], hepatocellular carcinoma [76], and gastric cancer [77], which are involved in inhibiting cell proliferation, cell cycle, and cell apoptosis. Furthermore, they found that the upregulation of MAPK1 was negatively regulated by miR-20a [75], miR-193b [76], and miR-378 [77], respectively. This suggests that MAPK1 vital gene plays essential roles in the development and progression of DN, which could participate in cell metabolism, cell proliferation, and apoptosis of podocyte, mesangial cell, etc. AKT1 is an important factor to regulate inflammation and apoptosis in kidney disease [78]. The phosphorylation of AKT1 can directly promote mesangial cell (MC) proliferation, basement membrane thickening, renal tubular epithelial cell transdifferentiation, and podocyte injury that are well known to play a critical role in the pathogenic mechanisms of DN [67, 79]. Evidence revealed that GSK3B is phosphorylated by AKT at serine 9, resulting in facilitating the expression of inflammatory cytokines [80]. Moreover, the inhibition of GSK3B induces human cell proliferation [81]. Kumar et al. found that AKT1 was one of the essential genes in the development and progression of SLE, with regard to increasing autoimmune responses via different signaling pathways [48]. TP53, a tumor suppressor, mediates glucose and lipotoxicity and causes a stop of the cell cycle, apoptosis, DNA repair, and metabolic changes, which is inhibited by microRNA-770-5p through regulating podocyte apoptosis [82]. This indicates that AKT1, GSK3B, TP53, and RELA might be associated with cell proliferation, cell cycle, and apoptosis of DN. The activation of JUN N-terminal kinase is associated with inflammatory factor secretion during DN [83]. Furthermore, JUN is a highly unstable protein due to polyubiquitination by GSK3 at Ser-243, and its level is inversely associated with GSK3 activity in mammalian cells that have entered the cell cycle [84], showing that JUN interacts with GSK3 via polyubiquitination. Nevertheless, the roles of JUN in DN require further research. Epidermal growth factor receptor (EGFR) belongs to the family of receptor tyrosine kinase ErbB receptors, and the activation of which can result in phosphorylation on specific tyrosine residues, such as ErbB2, ErbB3, or ErbB4, and further regulate cell proliferation, differentiation, and apoptosis [85]. The activation of EGFR leads to podocyte injury and loss in DN, while EGFR deletion in podocytes ameliorates glomerular injury and attenuates the progression of DN [86]. Previous studies also demonstrated that EGFR inhibition plays a nephroprotective role in DN via decreasing reactive oxygen species (ROS), inhibiting endoplasmic reticulum(ER) stress, alleviating podocyte injury, and increasing autophagy [87, 88]. RELA is one of the transcription factor genes in the NF-*κ*B signaling pathway that contributes to human disease processes, notably inflammatory diseases and cancer [89]. Site-specific phosphorylation has been proven to be a prerequisite for additional modifications regulating RELA activity, especially acetylation and ubiquitination [90]. A previous study showed that the phosphorylation of RELA at S536 may be related to regulating NF-kB activity, which was further corroborated in a mouse model that knocked down RELA S534A, a nonphosphorylatable mutant version of the human RELA S536 homolog in murine [91]. Besides, RELA also participates in the process of cell proliferation [92]. However, the role of RELA in DN remains unclear and requires further research.

**Figure 9:**
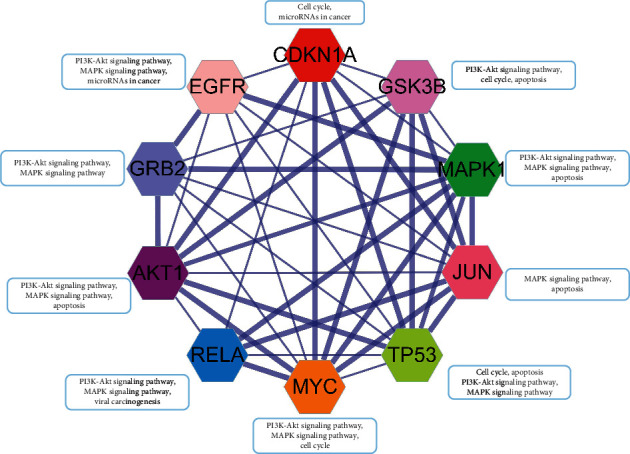
Interrelation analysis of the core genes MAPK1, AKT1, GSK3B, CDKN1A, TP53, RELA, MYC, GRB2, JUN, and EGFR related to *AR-PN* herb pair against DN. Genes are directly or indirectly involved in different pathways. The gene network was performed on the STRING and visualized in Cytoscape 3.8.0.

To explore the direct or indirect interactions between core target genes, an interrelation analysis for them was performed. As shown in Figure 9, the core target genes interacted with each other via multiple signaling pathways mainly associated with the PI3K-Akt signaling pathway, MAPK signaling pathway, cell cycle, apoptosis, microRNAs in cancer, and viral carcinogenesis. The core genes MAPK1, AKT1, TP53, RELA, MYC, GRB2, and EGFR are involved directly or indirectly in the PI3K-Akt signaling pathway regulating cell proliferation and MAPK signaling pathway resulting in apoptosis [93–96]. The cell cycle interacts with cell proliferation, differentiation, DNA damage, and cell apoptosis through TP53, GSK3B, CDKN1A, and MYC [97, 98]. Collectively, these findings indicate that the core genes could be critical factors for AR-PN herb pair against the development of DN, which require to be further experimentally validated to prove this hypothesis.

Molecular docking was performed to validate the binding interactions and affinity between active components (quercetin, kaempferol, isorhamnetin, and formononetin) and the core targets (MAPK1, AKT1, GSK3B, CDKN1A, TP53, RELA, MYC, GRB2, JUN, and EGFR). It has been demonstrated that a docking score between bioactive components and targets structures less than −7, −5, and −4.25 kcal·mol^−1^ represents a strong binding affinity, a good binding affinity, and binding affinity, respectively [99], which means the smaller the docking score, the more stable and stronger the affinity between components and targets (ligand and receptor) binding. As shown in Table 3, the binding energies of all docking are less than −5 kcal·mol^−1^, indicating that these target genes have great binding interactions and affinity with quercetin, kaempferol, formononetin, and isorhamnetin bioactive components. Moreover, the energy value of MAPK1 and JUN docked with quercetin, kaempferol, isorhamnetin, and formononetin was less than −8.0 kcal·mol^−1^ among the core target proteins. MAPK1 had the least binding affinity with quercetin, kaempferol, and formononetin at −8.4 kcal/mol. JUN docked with kaempferol and isorhamnetin had the lowest energy value at 9.7 kcal/mol. The docking results provide an efficient method to estimate the binding modes of *AR*-*PN* herb pair with DN-related core target proteins and provide us with sound directions for further validated.

Subsequently, ROC curves were applied to evaluate the diagnostic value of the core genes for DN. TP53, AKT1, RELA, and JUN with an AUC of 0.97436, 0.93162, 0.92308, and 0.86325, respectively, indicate that these hub genes exhibit high diagnostic values to distinguish DN patients and controls. Of course, their expression and related function also need to be further validated by mutation analysis, immune infiltration analysis, and RT-PCR.

Our study still has some limitations. Firstly, we only explored the potential functional mechanism of *AR*-*PN* herb pair on DN and have not identified the pharmacologically mutual interferences in bioactive components of *AR*-*PN* herb pair. Secondly, the DEGs of DN are acquired by analyzing published microarray data from the GEO database. Nevertheless, the sample size of DN-related DEGs is small, which needs to be enhanced in the future. Furthermore, the application of network pharmacology and bioinformatics in this study is just initial screening of the mechanisms and molecular targets of *AR*-*PN* herb pair against DN, and the results of this study have not been validated through experimental data. Hence, further studies, especially pharmacological experiments and clinical validations, are still required to be carried out to explain the complex bioactive chemical interactions. Moreover, we should provide larger groups and experimental evidence to validate these results with the interdisciplinary research works integrating computer science, network science, mathematics, and pharmacology in the future.

## 5. Conclusion

In summary, we utilized network pharmacology combined with bioinformatics to explore the mechanisms and molecular targets of *AR-PN* herb pair against DN in the present study. Our findings revealed that *AR-PN* herb pair exerts pharmacological effects on DN with various bioactive chemicals and the related pharmacological pathways, involving molecular targets and multiple cellulars, including antioxidant, anti-inflammatory, and antiapoptosis, and regulating the cell cycle. The *AR-PN* herb pair and its components may be promising drugs in the treatment of DN in the future.

## Figures and Tables

**Table 1:** Information of bioactive components of AR and PN.

Herb	Mol ID	Molecule name	OB (%)	DL
*AR*	MOL000211	Mairin	55.38	0.78
*AR*	MOL000239	Jaranol	50.83	0.29
*AR*	MOL000296	Hederagenin	36.91	0.75
*AR*	MOL000033	(3S,8S,9S,10R,13R,14S,17R)-10,13-Dimethyl-17-[(2R,5S)-5-propan-2-yloctan-2-yl]-2,3,4,7,8,9,11,12,14,15,16,17-dodecahydro-1H-cyclopenta[a]phenanthren-3-ol	36.23	0.78
*AR*	MOL000354	Isorhamnetin	49.6	0.31
*AR*	MOL000371	3,9-di-O-Methylnissolin	53.74	0.48
*AR*	MOL000374	5'-Hydroxyiso-muronulatol-2',5'-di-O-glucoside	41.72	0.69
*AR*	MOL000379	9,10-Dimethoxypterocarpan-3-O-beta-D-glucoside	36.74	0.92
*AR*	MOL000380	(6aR,11aR)-9,10-Dimethoxy-6a,11a-dihydro-6H-benzofurano[3,2-c]chromen-3-ol	64.26	0.42
*AR*	MOL000387	Bifendate	31.1	0.67
*AR*	MOL000392	Formononetin	69.67	0.21
*AR*	MOL000398	Isoflavanone	109.99	0.3
*AR*	MOL000417	Calycosin	47.75	0.24
*AR*	MOL000422	Kaempferol	41.88	0.24
*AR*	MOL000433	FA	68.96	0.71
*AR*	MOL000438	(3R)-3-(2-Hydroxy-3,4-dimethoxyphenyl)chroman-7-ol	67.67	0.26
*AR*	MOL000439	Isomucronulatol-7,2'-di-O-glucosiole	49.28	0.62
*AR*	MOL000442	1,7-Dihydroxy-3,9-dimethoxy pterocarpene	39.05	0.48
*PN*	MOL001792	DFV	32.76	0.18
*PN*	MOL001494	Mandenol	42	0.19
*PN*	MOL007475	Ginsenoside F2	36.43	0.25
*PN*	MOL002879	Diop	43.59	0.39
*PN*	MOL005344	Ginsenoside Rh2	36.32	0.56
*PN*	MOL000358	Beta-sitosterol	36.91	0.75
*PN*	MOL000449	Stigmasterol	43.83	0.76
*AR* and *PN*	MOL000098	Quercetin	46.43	0.28

*AR*: *Astragalus Radix*; *PN*: *Panax notoginseng*; OB: oral bioavailability; DL: druglikeness.

**Table 2:** The information of 115 candidate targets.

No.	Genes	Protein name	Betweenness centrality	Closeness centrality	Degree
1	NTRK1	Neurotrophic receptor tyrosine kinase 1	0.05711884	0.56600852	1199
2	TP53	Tumor protein p53	0.02958689	0.5339014	768
3	APP	Amyloid-beta precursor protein	0.04325808	0.53585828	746
4	CUL3	Cullin 3	0.01621362	0.52767084	744
5	EGFR	Epidermal growth factor receptor	0.03631785	0.53435673	738
6	MCM2	Minichromosome maintenance complex component 2	0.01374128	0.52022299	639
7	ESR1	Estrogen receptor 1	0.01715955	0.52376403	634
8	XPO1	Exportin 1	0.02022198	0.52090261	631
9	FN1	Fibronectin 1	0.01700008	0.51837844	612
10	UBC	Ubiquitin C	0.01612385	0.52282751	583
11	CDK2	Cyclin-dependent kinase 2	0.01028813	0.50905292	554
12	COPS5	COP9 signalosome subunit 5	0.00808418	0.51083159	517
13	CUL7	Cullin 7	0.0048695	0.50431183	515
14	HSP90AA1	Heat shock protein 90 alpha family class A member 1	0.0130468	0.5220807	510
15	GRB2	Growth factor receptor bound protein 2	0.01540288	0.51268264	485
16	RNF2	Ring finger protein 2	0.00618846	0.4979564	484
17	MYC	V-myc avian myelocytomatosis viral oncogene homolog	0.01293694	0.50705202	482
18	SIRT7	Sirtuin 7	0.00526073	0.49220065	470
19	CUL1	Cullin 1	0.00576927	0.50257821	469
20	YWHAZ	Tyrosine 3-monooxygenase/tryptophan 5-monooxygenase activation protein zeta	0.00881384	0.51503053	466
21	OBSL1	Obscurin-like 1	0.00370032	0.49442002	454
22	CAND1	Cullin-associated and neddylation dissociated 1	0.00443969	0.49280899	450
23	NPM1	Nucleophosmin	0.00555129	0.51059371	447
24	ITGA4	Integrin subunit alpha 4	0.00477184	0.49671574	444
25	VCP	Valosin-containing protein	0.00713559	0.51005931	413
26	CCDC8	Coiled-coil domain containing 8	0.00480319	0.49453151	412
28	EP300	E1A binding protein p300	0.00792101	0.50367478	410
27	HSP90AB1	Heat shock protein 90 alpha family class B member 1	0.00696006	0.51089109	410
29	VCAM1	Vascular cell adhesion molecule 1	0.0048008	0.4901654	404
30	CDC5L	Cell division cycle 5 like	0.00445741	0.4891268	399
31	FBXO6	F-box protein 6	0.00660866	0.4884731	393
32	BRCA1	BRCA1, DNA repair associated	0.00689339	0.49897611	391
33	TRAF6	TNF receptor-associated factor 6	0.00575486	0.49914647	378
34	HNRNPU	Heterogeneous nuclear ribonucleoprotein U	0.00359109	0.49694086	371
35	SNW1	SNW domain containing 1	0.00370573	0.49164892	370
36	HDAC1	Histone deacetylase 1	0.00504406	0.48798398	362
37	EED	Embryonic ectoderm development	0.00288746	0.47673913	359
38	HUWE1	HECT, UBA, and WWE domain containing 1, E3 ubiquitin protein ligase	0.00326087	0.49508974	358
39	EWSR1	EWS RNA binding protein 1	0.00617052	0.49931694	356
40	HNRNPA1	Heterogeneous nuclear ribonucleoprotein A1	0.00376562	0.49660326	352
41	MDM2	MDM2 proto-oncogene	0.0054897	0.49744811	346
42	UBE2I	Ubiquitin conjugating enzyme E2 I	0.00515795	0.4938077	344
43	YWHAQ	Tyrosine 3-monooxygenase/tryptophan 5-monooxygenase activation protein theta	0.00453158	0.49643463	338
47	RPA1	Replication protein A1	0.00447185	0.4904943	329
46	VHL	Von Hippel-Lindau tumor suppressor	0.0044112	0.49126344	329
44	PARK2	Parkin RBR E3 ubiquitin protein ligase	0.00331119	0.48923592	329
45	HSPA5	Heat shock protein family A (Hsp70) member 5	0.002573	0.50292398	329
48	HDAC5	Histone deacetylase 5	0.00203062	0.48282695	327
49	HIST1H3E	Histone cluster 1 H3 family member e	0.00117386	0.4791348	320
50	HIST1H3B	Histone cluster 1 H3 family member b	0.00117386	0.4791348	320
51	HIST1H3G	Histone cluster 1 H3 family member g	0.00117386	0.4791348	320
52	HIST1H3C	Histone cluster 1 H3 family member c	0.00117386	0.4791348	320
53	HIST1H3H	Histone cluster 1 H3 family member h	0.00117386	0.4791348	320
54	HIST1H3J	Histone cluster 1 H3 family member j	0.00117386	0.4791348	320
55	HIST1H3A	Histone cluster 1 H3 family member a	0.00117386	0.4791348	320
56	HIST1H3D	Histone cluster 1 H3 family member d	0.00117386	0.4791348	320
57	HIST1H3I	Histone cluster 1 H3 family member i	0.00117386	0.4791348	320
58	HIST1H3F	Histone cluster 1 H3 family member f	0.00117386	0.4791348	320
59	HSPB1	Heat shock protein family B (small) member 1	0.00950276	0.49475465	319
60	HSPA8	Heat shock protein family A (Hsp70) member 8	0.00598969	0.50361695	312
61	SRC	SRC proto-oncogene, non-receptor tyrosine kinase	0.00389245	0.50017106	312
62	RPA2	Replication protein A2	0.00253078	0.48961822	310
63	CUL2	Cullin 2	0.00217148	0.48171334	309
64	CTNNB1	Catenin beta 1	0.00495783	0.5011426	304
65	EEF1A1	Eukaryotic translation elongation factor 1 alpha 1	0.00334497	0.49660326	304
66	YWHAG	Tyrosine 3-monooxygenase/tryptophan 5-monooxygenase activation protein gamma	0.00349418	0.49570524	300
67	AKT1	AKT serine/threonine kinase 1	0.00665037	0.49682827	296
68	AR	Androgen receptor	0.00533699	0.49264293	294
70	CREBBP	CREB binding protein	0.00344521	0.47960634	292
69	U2AF2	U2 small nuclear RNA auxiliary factor 2	0.00188521	0.4802365	292
71	IKBKG	Inhibitor of nuclear factor kappa B kinase subunit gamma	0.00421812	0.49126344	291
72	MAPK1	Mitogen-activated protein kinase 1	0.00590832	0.493086	287
73	YWHAE	Tyrosine 3-monooxygenase/tryptophan 5-monooxygenase activation protein epsilon	0.00319492	0.49253229	283
74	PAN2	PAN2 poly(A) specific ribonuclease subunit	0.00154999	0.46525936	281
75	SUZ12	SUZ12 polycomb repressive complex 2 subunit	0.00168493	0.46605037	279
76	IKBKE	Inhibitor of nuclear factor kappa B kinase subunit epsilon	0.00234006	0.47493232	276
77	ARRB2	Arrestin beta 2	0.00295499	0.48351891	274
78	CUL5	Ccullin 5	0.00161801	0.47421343	274
79	FUS	FUS RNA binding protein	0.00199535	0.48673843	271
80	SMURF1	SMAD specific E3 ubiquitin protein ligase 1	0.00239525	0.48288011	270
81	RPA3	Replication protein A3	0.00151664	0.47575659	268
82	STAU1	Staufen double-stranded RNA binding protein 1	0.00121324	0.47908247	261
83	RELA	RELA proto-oncogene, NF-kB subunit	0.00499948	0.48700866	260
84	GSK3B	Glycogen synthase kinase 3 beta	0.00641109	0.48641455	256
85	SMAD3	SMAD family member 3	0.00358228	0.48956357	255
86	BMI1	BMI1 proto-oncogene, polycomb ring finger	0.00156804	0.46619898	254
87	COMMD3-BMI1	COMMD3-BMI1 readthrough	0.00156804	0.46619898	254
88	BAG3	BCL2-associated athanogene 3	0.00385077	0.47819451	252
89	YWHAB	Tyrosine 3-monooxygenase/tryptophan 5-monooxygenase activation protein beta	0.00260668	0.48722506	250
90	HSPA4	Heat shock protein family A (Hsp70) member 4	0.00289896	0.49548125	248
91	PARP1	Poly(ADP-ribose) polymerase 1	0.00322874	0.48086833	247
92	SMAD2	SMAD family member 2	0.00305262	0.48002627	246
93	RPS27A	Ribosomal protein S27a	0.00243271	0.4884731	246
94	FLNA	Filamin A	0.00324118	0.4884731	245
95	MYH9	Myosin heavy chain 9	0.00289354	0.48673843	244
96	CDKN1A	Cyclin-dependent kinase inhibitor 1A	0.00408751	0.48123766	241
98	ACTB	Actin beta	0.00348475	0.49325236	238
97	TARDBP	TAR DNA binding protein	9.56E-04	0.47436729	238
99	HDAC2	Histone deacetylase 2	0.00315062	0.47055037	236
100	ABL1	ABL proto-oncogene 1, nonreceptor tyrosine kinase	0.00149438	0.49076871	236
101	CUL4B	Cullin 4B	0.00369003	0.47788189	234
102	FYN	FYN proto-oncogene, Src family tyrosine kinase	0.00105834	0.46619898	234
103	EZH2	Enhancer of zeste 2 polycomb repressive complex 2 subunit	0.00157335	0.46884019	233
104	CRK	CRK proto-oncogene, adaptor protein	0.00664489	0.48218997	232
105	PRKCA	Protein kinase C alpha	0.00314723	0.47971125	232
107	ARRB1	Arrestin beta 1	0.00432736	0.48018393	231
106	JUN	Jun proto-oncogene, AP-1 transcription factor subunit	0.00304336	0.47762169	231
108	TUBB	Tubulin beta class I	0.00214432	0.49464306	231
109	HNRNPK	Heterogeneous nuclear ribonucleoprotein K	0.00139431	0.48787542	230
110	RPL10	Ribosomal protein L10	0.0011806	0.46314678	229
112	CLTC	Clathrin heavy chain	0.00232751	0.48652246	228
111	FBXW11	F-box and WD repeat domain containing 11	0.00221635	0.47298609	228
113	HLA-B	Major histocompatibility complex, class I, B	0.00338256	0.45549901	227
114	PRKDC	Protein kinase, DNA-activated, catalytic polypeptide	0.00185628	0.48950893	227
115	BTRC	Beta-transducin repeat-containing E3 ubiquitin protein ligase	0.00175312	0.47793397	227

**Table 3:** Docking scores of targets with components (kcal·mol^−1^).

Target name	PDBID	Components			
		Quercetin	Kaempferol	Isorhamnetin	Formononetin
MAPK1	4iz5	−8.4	−8.4	−8.4	−8
AKT1	1unq	−6.4	−6	−6.3	−6.6
GSK3B	4afj	−8.3	−8.4	−8.4	−7.6
CDKN1A	2zvw	−7.5	−7.5	−7.5	−7.4
TP53	2k8f	−6.9	−6.4	−6.7	−7.4
RELA	1nfi	−7.7	−8.5	−8.6	−8.3
MYC	5g1x	−7.9	−7.9	−7.5	−7.5
GRB2	1gri	−7.5	−7.6	−7.7	−7.6
JUN	1s9k	−8.6	−9.7	−9.7	−8.3
EGFR	5wb7	−7.7	−7.9	−8	−8

## Data Availability

The data used to support the findings of this study are included within the article.
